# Role of IL-33 and ST2 signalling pathway in multiple sclerosis: expression by oligodendrocytes and inhibition of myelination in central nervous system

**DOI:** 10.1186/s40478-016-0344-1

**Published:** 2016-07-26

**Authors:** Debbie Allan, Karen J. Fairlie-Clarke, Christina Elliott, Cornelia Schuh, Susan C. Barnett, Hans Lassmann, Christopher Linnington, Hui-Rong Jiang

**Affiliations:** 1 Strathclyde Institute of Pharmacy and Biomedical Sciences, University of Strathclyde, 161 Cathedral Street, Glasgow, UK; 2 Institute of Infection, Immunity and Inflammation, University of Glasgow, Scotland, UK; 3 Department of Neuroimmunology, Centre for Brain Research, Medical University Vienna, Vienna, Austria

**Keywords:** IL-33, ST2, Multiple sclerosis, Oligodendrocyte, Myelination

## Abstract

Recent research findings have provided convincing evidence indicating a role for Interleukin-33 (IL-33) signalling pathway in a number of central nervous system (CNS) diseases including multiple sclerosis (MS) and Alzheimer’s disease. However, the exact function of IL-33 molecule within the CNS under normal and pathological conditions is currently unknown. In this study, we have mapped cellular expression of IL-33 and its receptor ST2 by immunohistochemistry in the brain tissues of MS patients and appropriate controls; and investigated the functional significance of these findings in vitro using a myelinating culture system. Our results demonstrate that IL-33 is expressed by neurons, astrocytes and microglia as well as oligodendrocytes, while ST2 is expressed in the lesions by oligodendrocytes and within and around axons. Furthermore, the expression levels and patterns of IL-33 and ST2 in the lesions of acute and chronic MS patient brain samples are enhanced compared with the healthy brain tissues. Finally, our data using rat myelinating co-cultures suggest that IL-33 may play an important role in MS development by inhibiting CNS myelination.

**Electronic supplementary material:**

The online version of this article (doi:10.1186/s40478-016-0344-1) contains supplementary material, which is available to authorized users.

## Introduction

Signal transduction by Interleukin-33 (IL-33) is implicated in the pathogenesis of an increasing number of human diseases [1–3], in which it is generally regarded to act as an alarmin that alerts the immune system to necrotic cell injury and tissue damage [4–6]. This response involves a classical extracellular cytokine signalling pathway involving the heterodimer receptor complex ST2 and IL-1 receptor accessory protein (IL-1RAcP) [7], or acting as an intracellular nuclear factor that reduces pro-inflammatory signalling by sequestering NF-kB [8]. However, a more recent study by Kakkar et al. [9] reported that membrane bound vesicles containing IL-33 can be secreted by living cells, indicating a function beyond that of an endogenous danger signal. Although IL-33 is clearly an important immunomodulatory cytokine, there are intriguing hints to suggest that it may also play other roles, in particular in the central nervous system (CNS) where its expression is significantly high compared to other tissues [10].

This concept is supported by the important function of IL-33 in CNS development [11] and its association with a variety of neurological diseases [3, 12], including Alzheimer’s disease (AD) in which genetic variants of *Il-33* are associated with increased disease susceptibility [13, 14]. Amyotrophic lateral sclerosis is also shown to be associated with reduced serum IL-33 levels compared with healthy controls [15] which may reflect a corresponding increase in availability of soluble ST2 receptor. The role of IL-33 in inflammatory CNS diseases such as multiple sclerosis (MS) is of particular interest as MS is a disease characterised by immune-mediated demyelination of axons, thus IL-33 has the potential to modulate both the immune and the CNS system and therefore to influence disease pathology. This is supported by recent findings of increased expression of IL-33 in the periphery and CNS tissues of MS patients [16, 17]. However the pathophysiological significance of these observations remains obscure, studies on experimental autoimmune encephalomyelitis (EAE) provide contradictory findings as to the role of IL-33 in neuroinflammatory disease. Ablation of IL-33 signalling by deleting its receptor in mice resulted in exacerbation of EAE [18, 19], whilst utilising an IL-33 blocking antibody resulted in the converse effect, inhibiting disease onset and reducing its severity [20]. The reason for this dichotomy remains unknown but may reflect an unexpected role for IL-33 within the CNS compartment, above and beyond its ability to act as an immunomodulatory cytokine.

Surprisingly despite increased evidence supporting a role for IL-33 in a variety of CNS diseases, its function within the CNS under normal and pathological conditions is unknown. As a first step towards resolving these questions we determined cellular expression of IL-33 and ST2 by immunohistochemistry in the brain tissues of MS patients together with appropriate controls. Following that we investigated the function of IL-33/ST2 signaling pathway in CNS using rat CNS myelinating co-cultures.

## Materials and Methods

### Antibodies

The following primary antibodies were used for human brain tissues: anti-IL-33 (Enzo Lifescience), anti-ST2 (Sigma-Aldrich), anti-GFAP (DAKO), and anti-Iba1 (Wako). Antibodies against SMI-31 and CA-II were purchased from Abcam. Primary antibodies for immunolabelling cells within the myelinating cultures include: anti-ST2 (Sigma-Aldrich), anti-GFAP (DAKO), anti-SMI-31 (Abcam), anti-MBP (Chemicon). The antibody O4 [21], and other anti-NeuN and anti-Olig2 antibodies were purchased from Millipore. All the primary antibodies were tested and an optimal dilution of 1:100 of the original purchased stock was used in staining except CA-II was diluted 1:500. Appropriate isotype control antibodies, biotinylated antibodies and fluroscence conjugated antibodies were purchased from Sigma-Aldrich, DAKO, R&D Systems or Jackson Immunoresearch.

### MS patient specimens

Archived formalin-fixed, paraffin-embedded brain materials from 14 MS patients and 6 controls without neurological disease or evidence of brain lesions were used in this study. Samples from MS patients have been extensively characterised in the Center for Brain Research of the Medical University of Vienna. The samples include: 7 acute MS patients (Marburg’s type) with 2 females and an average age of 49.4 years, these cases all died within one year after disease onset and were selected because of the abundance of active MS lesions; 7 chronic MS patients with 4 females and an average age of 63.1 years, all with a clinical course of secondary progressive MS, these cases were selected on the presence of large numbers of slowly expanding and inactive chronic lesions; 6 healthy controls with 4 female and an average age of 58.7 years. Immunohistochemical staining was performed on the brain samples of all the patients and controls. Images between samples in each group were compared and verified by MS pathologists, representative images of consistent data in each group were presented. The study was approved by the ethics committee of the Medical University of Vienna (EK Nr: 078/11/2015).

### Immunohistochemical staining

Paraffin slides were heated in an oven at 60 °C for 35 min to soften the wax. The slides were then deparaffinised and hydrated through histoclear and several graded ethanol solutions. The sections were then rinsed in distilled water for 5 mins. To quench the endogenous peroxidase activity, the tissues were incubated in 0.5 % hydrogen peroxidase in methanol. The slides were then washed in Tris-buffered saline (TBS) and incubated with sodium citrate in a pressure cooker for antigen retrieval. Following that, tissues were washed and incubated with primary antibodies against IL-33 or ST2 overnight at 4°C. The following day, the slides were washed in TBS and incubated with the appropriate biotinylated antibodies for 1 h at room temperature (RT). The slides were then washed and incubated with horseradish peroxidase (HRP, Sigma-Aldrich) for 1 h at RT. After the HRP was washed off, the staining was visualised using Impact DAB solution (Vector Laboratories), the reaction was stopped with tap water. The slides were then counter stained using haematoxylin, dehydrated through graded ethanol alcohol solutions and mounted in DPX (Sigma-Aldrich).

Double immunohistochemical staining was performed by repeating the above procedure however using alkaline phosphatase in place of HRP and visualising with Vector ^®^ blue (Vector Laboratories) for the second primary antibody specific proteins. For fluorescence staining, FITC or TRITC-conjugated secondary antibodies were added to the tissue sections following incubation with the primary antibodies. Fluorescence staining sections were mounted with Vectashield containing DAPI (Vector Laboratories). Isotypes with matching IgG were used as negative controls for all the immunohistochemical staining.

### Myelinationing co-cultures

The protocol of generating myelinating spinal cord cultures has been previously described in by Sorensen et al. [22] and reported in some of our recent papers [23, 24]. Briefly neurospheres were derived from the corpus striatum of P1 Sprague Dawley rats and cultured in neurosphere medium (NSM) supplemented with 20 ng/ml of mouse sub maxillary gland epidermal growth factor (EGF, R & D Systems) to promote sphere formation. After 7 days in culture, neurospheres were carefully collected and transferred to 24 well plates with poly-L-lysine coated coverslips inside each well (~50,000 cells/coverslip), and cultured in Dulbecco’s Modified Eagle Medium (DMEM) with 10 % fetal bovine serum. The cell culture was routinely fed by removing half of the medium and replacing with fresh medium. It typically took 7 days before the astrocytes formed a confluent monolayer on the coverslip. Coverslips supporting the astrocytes were then placed in a small petri-dish before adding dissociated rat embryonic spinal cord cells.

To obtain the dissociated rat spinal cord cells, E15.5 embryos were obtained from time mated Sprague Dawley female rats. The cranial 5- to 6- mm sections of spinal cord from the embryos were dissected and stripped of meninges. Tissues were then dissociated with trypsin and collagenase, and plated onto coverslips prepared in the dish with a density of 150,000 cells/50 μl per coverslip. The cells were left to attach for 2 h and then 1 ml of differentiation medium, which was DMEM containing 4500mg/ml glucose, 10ng/ml biotin and 0.5 % N1 hormone mixture [1mg/ml apotranferrin, 20mM putrescine, 4μM progesterone, 6μm selenium, 50nM hydrocortisone and 0.5 mg/ml insulin (Sigma-Aldrich, UK)] was added. The cultures were fed regularly three times a week by replacing half of the medium with fresh differentiation medium. After 12 days culture in vitro (DIV), insulin was removed from the culture medium to promote myelination. The cultures were maintained for a further period of 14–16 days. From DIV 18 -28, some myelinating culture cells were fed three times a week by replacing 500 μl of medium with equal amount of fresh medium containing recombinant IL-33 (final concentration 25 ng/ml or 100 ng/ml).

### Staining and analysis of myelination in the co-cultures

Cells were fixed with 4 % paraformaldehyde for 20 mins, washed, and followed by permeabilision with 0.5 % Triton X-100 for 15 mins at RT. Cells were incubated overnight at 4 °C with specific primary antibodies against ST2, SMI-31 (neurofilament marker to visualise axons), MBP (myelin) or Olig2. On the following day, primary antibodies were removed and coverslips were washed with PBS thoroughly before appropriate fluorochrome conjugated secondary antibodies were added and incubated for a further 15 mins. Coverslips were then washed and mounted to glass slides in Vectashield (Vector laboratories).

All images taken for analysis of cell number, morphology and axon/myelin qualification were obtained using an Olympus BX51 fluoroscent microscope. For myelin analysis, a minimum of 30 images (x10 magnification) were taken at random with 10 images per coverslip for each sample. The images were analysed using the software Image J. Using this software each image was separated into three channels i.e. blue, red and green. The axonal density was determined by calculating the percentage of SMI-31+ pixels compared to the total number of pixels within the image (also as field). The percentage of myelinated axons was quantified by placing a transparent layer on top of the image in Adobe Photoshop® and drawing blue lines over the MBP+ myelin (green) only focusing on the sheaths. The myelin was then quantified by using a macro generated by the group using Image J. Myelination was expressed as the total number of myelin pixels / total number of SMI-31 pixels. Data reported here were compiled from 3 independent experiments and analysed in the statistical package JMP8.0 using linear models. Percentage of axonal density and percentage of myelinated axons were analysed to determine changes over time and after the addition of IL-33 at 28 DIV. Significant differences between groups were determined by Student’s t-test, *p* < 0.05 was considered to be significant different between groups.

## Results

### Altered expression level and pattern of IL-33 in lesions of MS patient brain tissues

**Fig. 1:**
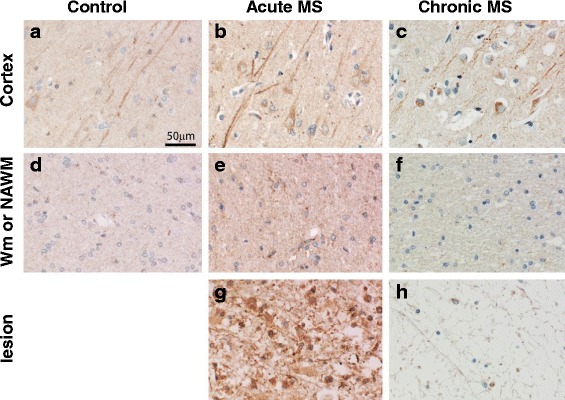
Expression of IL-33 (*brown*) in healthy and MS brain patient samples. Immunohistochemical staining was used to determine the expression of IL-33 in the cortex and WM of healthy brain samples, and in the cortex, NAWM and lesion of acute and chronic MS patients. **a** cortex and **d** white matter of healthy brain tissues; **b** cortex, **e** NAWM and **g** lesion of acute MS brain sample; **c** cortex, **f** NAWM and **h** lesion of chronic MS brain sample

To determine the expression of IL-33 in MS lesions, immunohistochemical staining was performed in human brain tissues from all MS patients and controls as described in Materials and Methods. IL-33 specificity was tested and confirmed using an isotype control antibody in human lung and brain tissues (Additional file 1: Figure S1A). Our data show that IL-33 was expressed in both the cortex and white matter (WM) of healthy controls (Fig. 1a, d). Expression of IL-33 in the cortex and normal appearing WM (NAWM) from chronic MS patients was similar to tissues of controls (Fig. 1c, f). IL-33 reactivity was seen in the cortex in granular form in a subset of neurons, including their dendrites and in some glia cells in the cortex and the white matter. However in cortex, NAWM and lesions of acute MS patients (Fig. 1b, e, g), IL-33 staining was of higher intensity than the control and chronic patient tissues. This was particularly evident in active lesions, where intense reactivity was present in glia cells and macrophages. In chronic (inactive) MS lesions, IL-33 reactivity was low and found in few glia cells and occasionally in axons (Fig. 1h).

#### Expression of IL-33 by axons, microglia and astrocytes

**Fig. 2:**
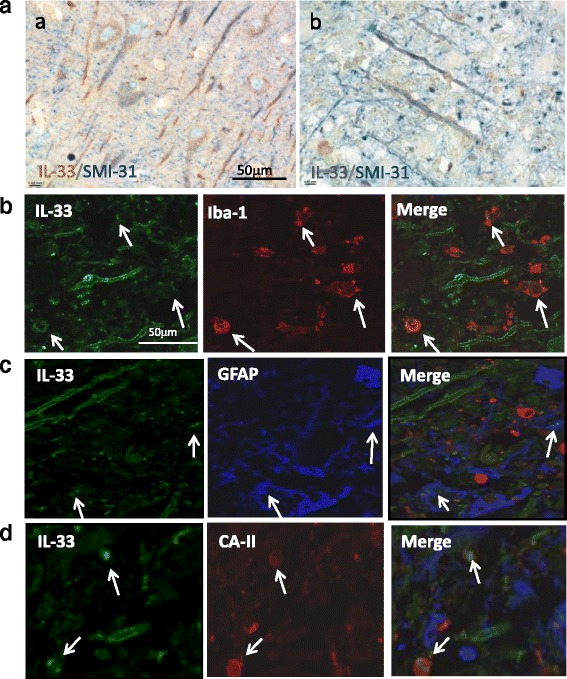
Expression of IL-33 by CNS resident cells in human. **a** Co-staining of IL-33 (*brown*) with SMI-31 (*blue*), a marker for neuron and axons, in cortex of normal human brain (*a*) and MS acute lesion (*b*). **b** Co-staining of IL-33 with Iba1, a marker for microglia cells, in human brain samples. *Arrows* indicate colocalization of IL-33 with Iba-1. **c** Co-staining of IL-33 with GFAP, a marker for astrocytes (*arrows*) in human brain samples. **d** Co-staining of IL-33 with CA-II, a marker for oligodendrocytes (*arrows*) in human brain samples

Co-localisation of IL-33 with CNS resident cells was investigated next. Double colour staining revealed that IL-33 expression in the cortex of healthy controls (Fig. 2a*a*) and MS patients (Fig. 2a*b*) was associated with neurons and axons as determined by co-localisation of IL-33 with the neurofilament marker SMI-31 (Fig. 2a). We further performed double or triple fluorescence staining to determine the expression of IL-33 by CNS glia cells, and demonstrate that some but not all Iba1 positive microglia (Fig. 2b, arrows) or GFAP positive astrocytes (Fig. 2c, arrows) express IL-33. Interestingly, IL-33 is also co-localised with CA-II, a marker for oligodendrocytes (Fig. 2d, arrows), indicating a potential role of IL-33 in demyelination or remyelination process in MS disease.

#### Expression of ST2 in the brain of MS patients and controls

**Fig. 3:**
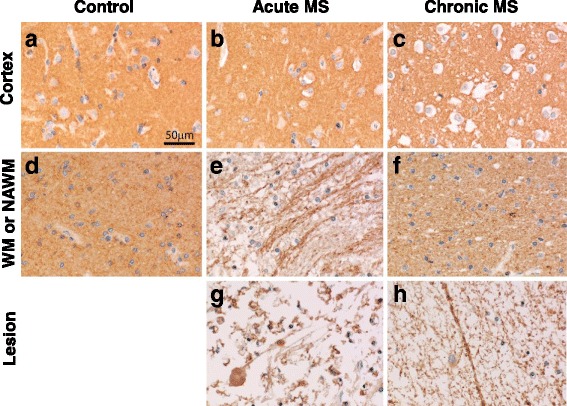
Expression of ST2 (*brown*) in healthy and MS patient brain samples. Immunohistochemical staining was used to determine the expression of ST2 in the cortex and WM of healthy brain samples, and in the cortex, NAWM and lesion of acute and chronic MS patients. **a** cortex and **d** white matter of healthy brain tissues; **b** cortex, **e** NAWM and **g** lesion of acute MS brain sample; **c** cortex, **f** NAWM and **h** lesion of chronic MS brain sample

Expression of ST2 in MS patient and control brain tissues was also examined using immunohistochemistry. ST2 expression in the cortex of both control and MS brain tissues was associated with a diffuse pattern of staining (Fig. 3a-f), suggesting a similar degree of expression of this receptor on all neurons and glia. It is worth noting that such staining was not observed when a matching isotype control antibody was used and specific staining of ST2 was observed in human lung tissues with the same ST2 antibody (Additional file 1: Figure S1B). A similar diffuse immunoreactivity was also seen in the NAWM of chronic MS patients (Fig. 3f) and WM of control tissues (Fig. 3d). However, at the edge of active lesions in acute MS (Fig. 3e) and in the demyelinated center of the lesions (Fig. 3h) accentuated immunoreactivity on myelinated nerve fibers was seen, possibly reflecting increased ST2 expression or accessibility on partly damaged myelin. In addition we found ST2 reactivity in macrophages within active lesions and in some axonal end bulbs at sites of axonal transection (Fig. 3g).

#### Expression of ST2 by axon and oligogendrocytes

**Fig. 4:**
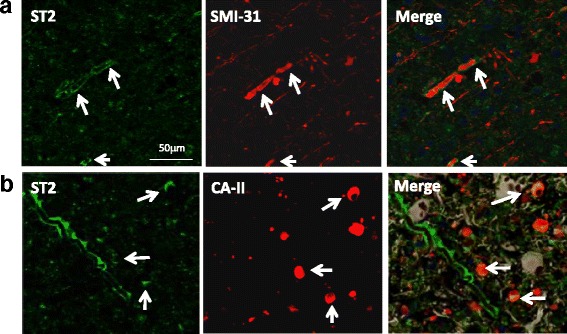
Expression of ST2 by CNS cells in human. **a** Co-staining of ST2 (*green*) with SMI-31 (*red*), a marker for axons, in human brain samples. *Arrows* indicate expression of ST2 around SMI-31 expressing axons. **b** Co-staining of ST2 (*green*) with CA-II (*red*) positive oligodendrocytes in human brain samples. *Arrows* indicate colocalization of ST2 with CA-II expressing oligodendrocytes

To clarify the role of IL-33/ST2 axis in the CNS, it is important to identify the ST2 positive CNS resident cells. Our dual staining of ST2 with SMI-31 revealed an expression of ST2 around axons in MS lesions (Fig. 4a). Further fluorescence staining experiment revealed that ST2 was expressed by some oligodendrocytes as determined by co-localisation of ST2 with CA-II (Fig. 4b). Thus our data here suggest a potential role for IL-33/ST2 signalling in the demyelination and/or remyelination process in CNS degenerative diseases such as MS.

#### A role for IL-33/ ST2 signalling in myelination

Expression of ST2 around damaged axons and by oligodendrocytes in CNS lesions of MS patients (Fig. 4) led to our investigation of whether myelination is affected by IL-33/ ST2 signalling. A well established rat CNS myelinating co-cultures was generated as described previously [22–24] and cells were treated with recombinant IL-33, after 16 days the number of oligodendrocytes, density of axons and myelinated axons was assessed. Our results show that the number of oligodendrocytes in the culture, indicated by Olig2 staining, increased significantly after 12 DIV in accordance with the removal of insulin from the culture media which inhibited myelination prior to this point (Additional file 2: Figure S2). Similar increase was also observed with MBP positive staining (Additional file 3: Figure S3) while the increase of SMI-31 expression (i.e. axons) was not significant.

**Fig. 5:**
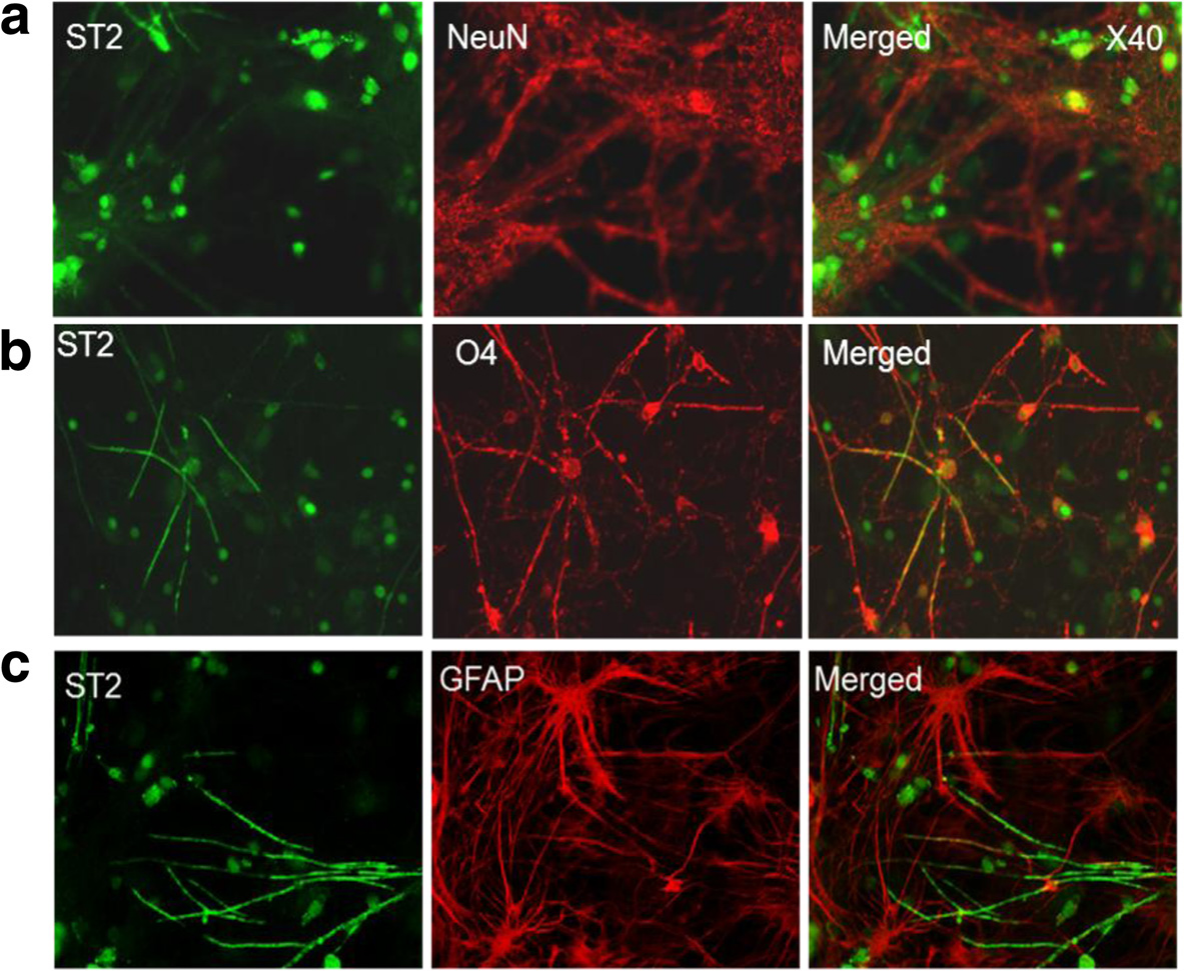
Expression of ST2 by CNS cells in rat melinating co-cultures. Double immunofluorescence staining of ST2 (*green*) and antibodies against NeuN (*red*, marker for neuron cells) and GFAP (*red*, astrocyte marker), and the O4 antibody (*red*, a marker for immature oligodendrocytes and mature progenitor cells) in the cultured CNS cells at 12 DIV

**Fig. 6:**
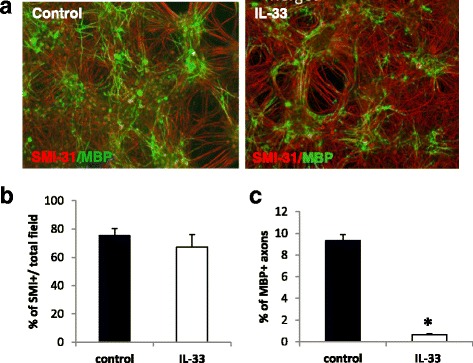
Recombinant IL-33 inhibits axon myelination in rat myelinating co-cultures. The cell cultures were treated with or without recombinant IL-33 from DIV 12 to 28, **a** and the cells immuno-labelled with SMI-31 and anti-MBP, markers for axons and mature myelin respectively. **b** Graph depicting the percentage of SMI-31+ cells in total field of view. **c** Graph depicting the percentage of myelination measured by MBP+ and SMI-31+ overlapping pixels/total SMI31 pixels. * *P* < 0.05. Data are presented as Mean + SEM, and were compiled from three independent experiments

To determine whether IL-33 receptor ST2 was expressed by the cultured CNS cells, we carried out a series of immunohistochemical staining and our results confirmed that ST2 is expressed by some neurons (Fig. 5a) and oligodendrocytes (Fig. 5b) but not astrocytes (Fig. 5c), which agrees with our in situ staining of ST2 by oligodendrocytes in human brain tissues. To test the effect of IL-33 on myelination, 25ng/ml or 100 ng/ml recombinant IL-33 was added to the culture at 12 DIV and similar effect was observed between the two groups. Our results show that there was no significant difference between the axonal densities of control and IL-33 treated cultures at 28 DIV as shown by the percentage of SMI-31+ axons (Fig. 6a and b), indicating that IL-33 does not impact the growth or viability of neurons and axonal formation. Surprisingly treatment with IL-33 resulted in a significant reduction in the proportion of myelinated axons (Fig. 6a and c) in the myelinating co-cultures, suggesting that IL-33 may have an inhibitory effect on myelination.

## Discussion

Despite recent research interest in the role of IL-33 in CNS diseases, the precise cellular source and expression levels of IL-33 and ST2 *in-situ* in human have not been conclusively determined. Our study here demonstrates that IL-33 protein is expressed by various CNS resident cells including neurons, astrocytes, oligodendrocytes and microglia cells, while its receptor ST2 is predominantly expressed by neurons and oligodendrocytes. In addition, the expression levels and patterns of IL-33 and ST2 in the lesions of acute and chronic MS patient brain samples were enhanced compared with the healthy brain tissues. Our study using rat myelinating co-cultures further revealed that IL-33 inhibits CNS myelination, thus suggesting how it may contribute to MS pathology.

Numerous studies have attempted to identify the expression of IL-33 and ST2 in CNS cells, however the results are confusing as some studies used in vitro cultured CNS cells, and others mainly focused on glial cells such as astrocytes [25–27]. Nevertheless findings from these studies have indicated that astrocytes are an important potential source of IL-33 in both mouse and human [16, 18, 25, 26], and that IL-33 released by astrocytes activates microglia cells during CNS diseases [25]. Our study here using *in situ* immunohistochemical staining confirms the co-localisation of IL-33 with GFAP in human brain tissues. Furthermore, we have revealed that astrocytes are not the exclusive source of IL-33 as our data demonstrate that IL-33 is also expressed by neurons. The expression of IL-33 by microglia cells remains controversial [26, 28]. Our data clearly indicate a co-localisation of IL-33 with some Iba1 expressing cells, suggesting a potential source of IL-33 by microglia cells, as proposed by Xiong et al. in AD [29].

While most research focus has been on IL-33, the expression of ST2 by CNS cells is less clear. ST2 mRNA was detected in cultured murine microglia and astrocyte cells [26]. As IL-33 is well known to induce the polarisation of alternatively activated (M2-like) macrophages in diseases such as asthma [30] and obesity [31], and M2-like microglia cells contribute to neuroprotection [32], expression of ST2 by microglia cells therefore may suggest that IL-33 plays an important role in MS development through modulating the polarisation of microglia cells in the CNS. Our *in situ* staining data here show that ST2 is accentuated around SMI-31 labelled axons and CA-II labelled oligodendendrocytes in human brains. The data with cross expression of IL-33 and ST2 by various CNS cells suggest complex autocrine and paracrine mechanisms of IL-33/ST2 signalling in the CNS compartment.

It has been well documented that IL-33 is a pleiotropic cytokine [10, 33–35] in regulating immune responses in various immune mediated diseases [36–38], and systemic administration of recombinant IL-33 to EAE mice after disease onset induces type 2 immune responses and reduces CNS inflammation [18]. The apparent up-regulation of IL-33 and ST2 at the acute lesions of MS patients indicates that IL-33 signalling is enhanced in CNS inflammation, thus suggesting a role for IL-33 in the pathogenesis of MS disease. Furthermore, the expression of both IL-33 and ST2 by various CNS cells indicates that IL-33/ST2 is likely to have its unique CNS specific function in addition to its immunomodulatory roles during CNS inflammation. However, its precise function in the CNS under normal and disease conditions remains poorly understood. Most current studies suggest that glial cell-released IL-33 activates neighbouring cells to produce inflammatory molecules, which further impact on the neuronal function in CNS, being either protective [5] or pathogenic [26, 29] under different disease conditions. The implication of *Il-3*3 gene polymorphism in the etiology of AD disease [13, 14] had led to a further investigation [29], which reported that IL-33 and ST2 positive cells were significantly increased in the AD brains when compared with non-AD brains, and the expression was associated with signatures of AD pathology (e.g. amyloid plagues and neurofibrillary tangles). The authors proposed that IL-33/ST2 axis may play an important role in AD pathogenesis via inducing inflammatory molecules released from the glial cells. In our study, while only a small increase of IL-33 expression was observed in the NAWM of MS patients, its levels were dramatically enhanced in active lesions of MS, which is in good agreement with previous findings of increased IL-33 mRNA at the lesion of MS patients by Christophi et al. [16]. While less is known about the expression of ST2 in the CNS, our data clearly show that the diffuse expression of ST2 in normal human cortex changed to an accentuated staining in the MS lesions in axons and in potentially damaged myelin, confirming a role for IL-33/ST2 pathway in MS disease.

Whether IL-33 has detrimental or beneficial effect on MS development and what are the underlying mechanisms are yet to be determined. Kempuraj et al [39] suggested the involvement of IL-33 in neurodegeneration and neuronal death as incubation of mixed astrocytes and neurons or neuronal culture with IL-33 reduced the number of microtubule-associated protein-2-immunoreactive cells. While several other studies confirmed the expression of IL-33 by murine oligodendrocytes [5, 11, 40], our observation of IL-33 and ST2 expression by oligodendrocytes in human CNS tissues support a role for IL-33/ST2 in CNS demyelination or remyelination. In order to understand the effect of IL-33 on CNS myelination, we used well-established rat CNS myelinating co-cultures. While IL-33 was shown to have no effect on the density of axons, our study show for the first time that IL-33 was able to inhibit axon myelination significantly. Whether IL-33 contributes to the initial demyelinating step, or is also involved in the following neurodegeneration in vivo remains unclear and merits further in-depth investigation. It is worth noted that the data were obtained using a rat myelinating culture system, our preliminary data from a mouse myelinating culture system showed no statistical significance of axon myelination with the presence of IL-33 in the culture (data not shown). It will need further investigation to understand whether this is a real species-specific difference or culture condition difference as the rat CNS culture system requires a pre-prepared single layer of astrocytes, which is not needed in the mouse culture.

Although the myelinating cultures do not mimic the complex in vivo model of demyelinating diseases, our data indicate the importance of IL-33/ST2 axis in MS development, possibly via its involvement in myelination process in CNS, in contrast to the protective anti-inflammatory function of recombinant IL-33 [18] and spinal cord-released IL-33 [28] in EAE. These findings however indicate the complex interplay between the CNS and immune system, and the different roles many cytokines play, e.g. TNF-α not only regulates inflammation but also performs a distinct set of other functions within the CNS compartment [41]. It is therefore important to fully define the roles of IL-33 in both systems before considering it as a new therapeutic target or reagent for MS disease.

## Conclusions

Together, the present study demonstrates that IL-33 and ST2 are highly expressed by various CNS resident cells, and there was a change of the expression levels and patterns of both molecules in the CNS lesions of MS patients compared with healthy brain tissues. Furthermore, IL-33/ST2 signalling pathway may have its unique important function in the myelination process in the CNS compartment, thus contributing to MS disease development.

## Abbreviations

AD, alzheimer’s disease; CNS, central nervous system; EAE, experimental autoimmune encephalomyelitis; IL-33, interleukin-33; MS, multiple sclerosis.

## Additional files

Additional file 1: Figure S1. Immunohistochemical staining of IL-33 and ST2 in human lung tissues. (A) IL-33 matched isotype control antibody staining in human lung (a) and brain (b) samples, and IL-33 staining in human lung sample (c and d). (B) ST2 matched isotype control antibody staining in human lung (a) and brain (b) tissues; ST2 staining in human lung sample (c and d). (a, b and c), x10 magnification; d. x25 magnification. (PDF 215 kb) Additional file 2: Figure S2. The number of oligodendrocytes in the rat CNS myelinating co-cultures. (A) Percentage of Olig2+ cells in total field. (B) Images of Olig2 staining in the culture system at DIV 12, 18 and 28. Data are presented as Mean + SEM, and were compiled from three independent experiments. (PDF 189 kb) Additional file 3: Figure S3. The number of axons and myelinated axons at DIV 12, 18 and 28 of the rat CNS myelinating culture. (A) Percentage of SMI-31+ cells in view field; (B) Percentage of MBP+ axons in view filed. (C) Representative images of SMI-31 and MBP staining in the myelinating culture at DIV 12, 18 and 28. Data are presented as Mean + SEM, and were compiled from three independent experiments. (DOC 356 kb)
