# Supplementary figures and images for: Role of IL-33 and ST2 signalling pathway in multiple sclerosis: expression by oligodendrocytes and inhibition of myelination in central nervous system
Source: Acta Neuropathol Commun. 2016 Jul 26;4:75. doi: 10.1186/s40478-016-0344-1 (PMC4960877; doi:10.1186/s40478-016-0344-1)

## Supplement Figure 1

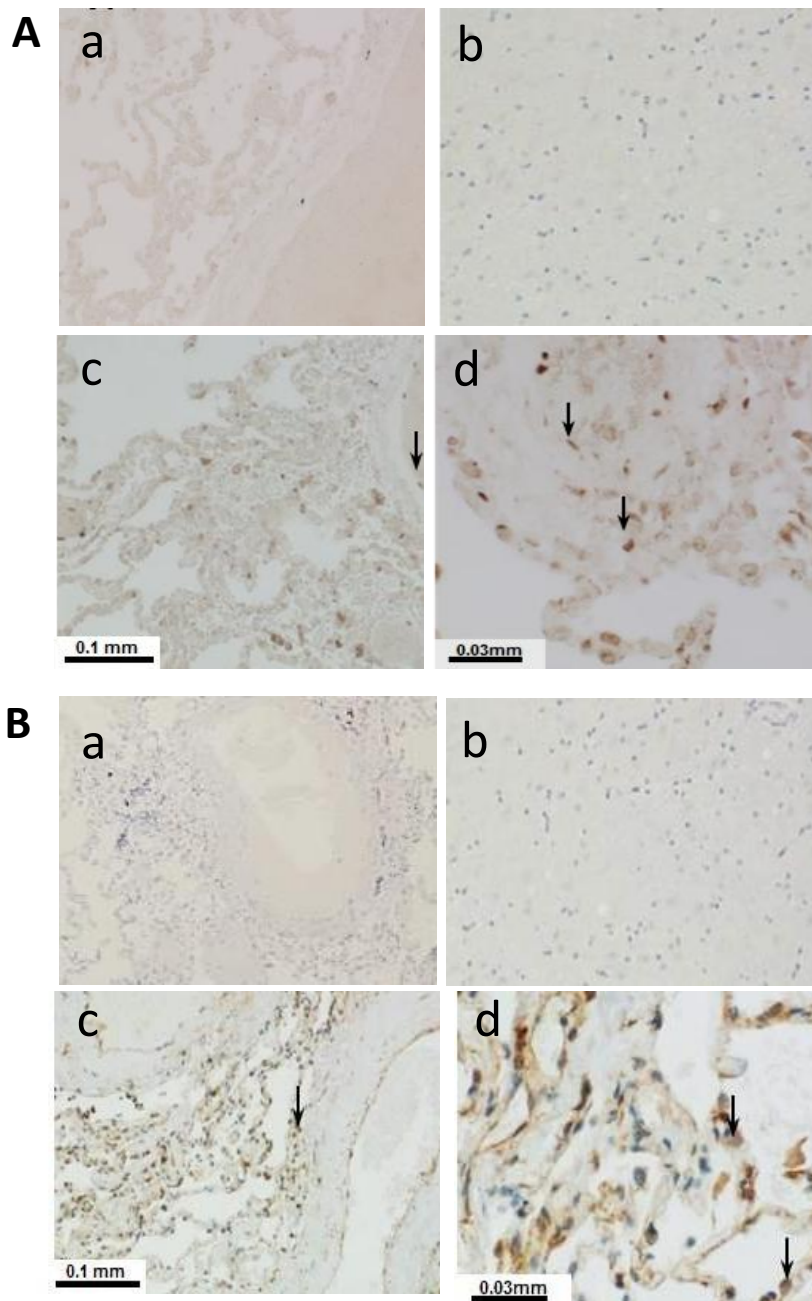

Supplement: Additional file 1: Figure S1. — Immunohistochemical staining of IL-33 and ST2 in human lung tissues. (A) IL-33 matched isotype control antibody staining in human lung (a) and brain (b) samples, and IL-33 staining in human lung sample (c and d). (B) ST2 matched isotype control antibody staining in human lung (a) and brain (b) tissues; ST2 staining in human lung sample (c and d). (a, b and c), x10 magnification; d. x25 magnification. (PDF 215 kb) [file 40478_2016_344_MOESM1_ESM.pdf]

Supplement Figure 2

A

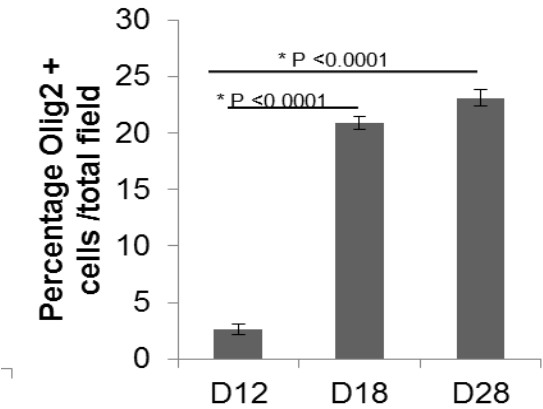

B

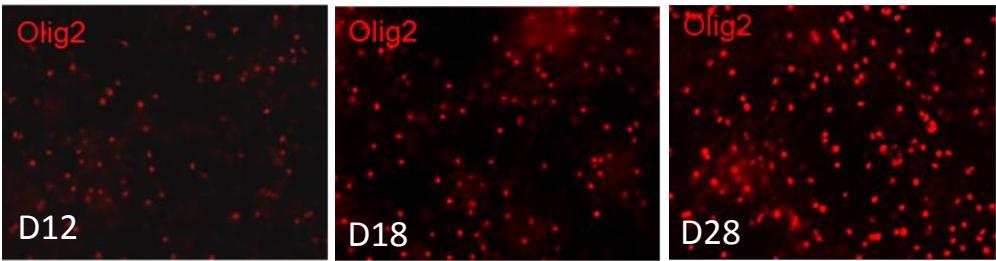

Supplement: Additional file 2: Figure S2. — The number of oligodendrocytes in the rat CNS myelinating co-cultures. (A) Percentage of Olig2+ cells in total field. (B) Images of Olig2 staining in the culture system at DIV 12, 18 and 28. Data are presented as Mean + SEM, and were compiled from three independent experiments. (PDF 189 kb) [file 40478_2016_344_MOESM2_ESM.pdf]
