# Supplementary material for: Role of IL-33 and ST2 signalling pathway in multiple sclerosis: expression by oligodendrocytes and inhibition of myelination in central nervous system
Source: Acta Neuropathol Commun. 2016 Jul 26;4:75. doi: 10.1186/s40478-016-0344-1 (PMC4960877; doi:10.1186/s40478-016-0344-1)

Supplement Figure 3

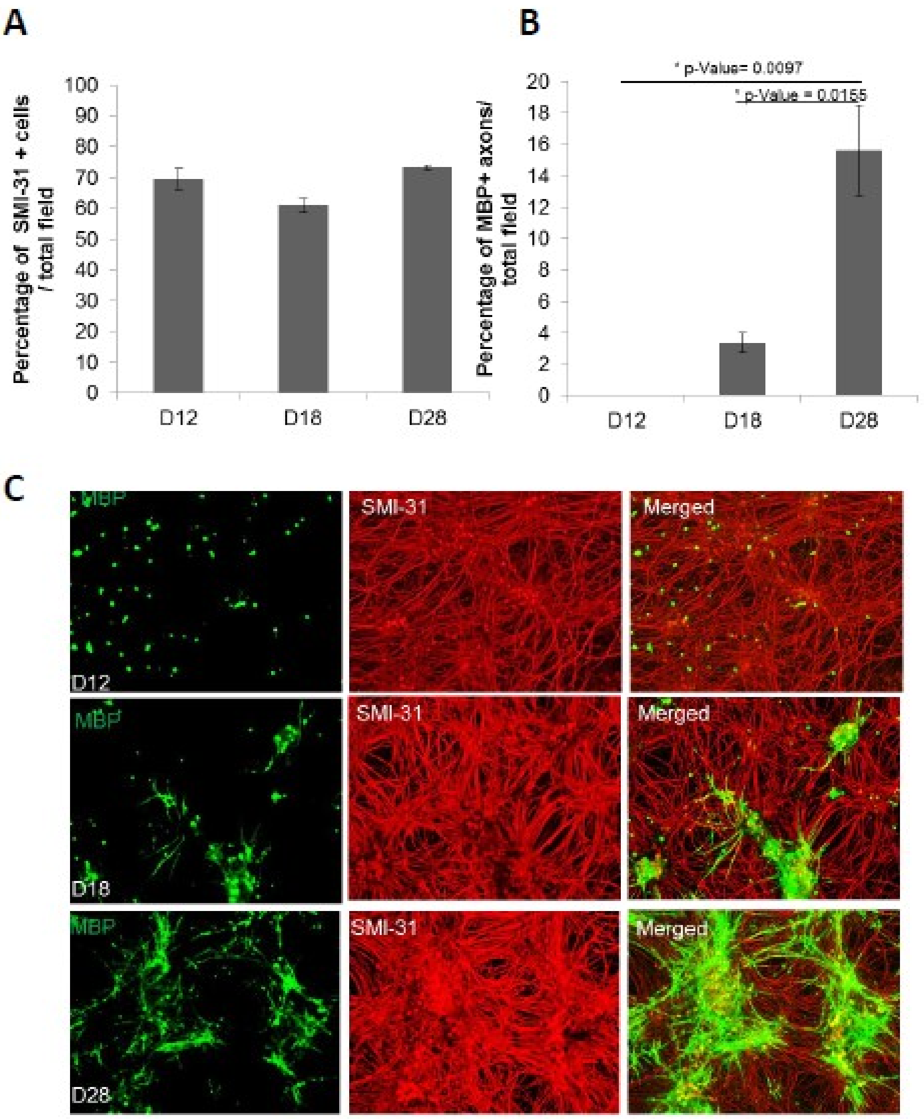

Supplement: Supplementary file 2 [file 40478_2016_344_MOESM3_ESM.doc]
